# Integrating Remote Sensing and Geophysics for Exploring Early Nomadic Funerary Architecture in the “Siberian Valley of the Kings”

**DOI:** 10.3390/s19143074

**Published:** 2019-07-11

**Authors:** Gino Caspari, Timur Sadykov, Jegor Blochin, Manuel Buess, Matthias Nieberle, Timo Balz

**Affiliations:** 1 Department of Archaeology, University of Sydney, The Quadrangle A14, 2006 Sydney, Australia; 2 Institute of Archaeological Sciences, University of Bern, Mittelstrasse 43, 3012 Bern, Switzerland; 3 Institute for the History of Material Culture, Russian Academy of Sciences, Dvortsovaya nabereznaya 18, 191186 St. Petersburg, Russia; 4 Cantonal Archaeology Aargau, Industriestrasse 3, 5200 Brugg, Switzerland; 5 Archaeological Institute, University of Cologne, Albertus-Magnus-Platz, 50923 Cologne, Germany; 6 State Key Laboratory of Information Engineering in Surveying, Mapping and Remote Sensing, Wuhan University, Luoyu Road 129, Wuhan 430079, China

**Keywords:** early nomadic, Scythian, funerary architecture, burial mound, Early Iron Age, steppe, Siberia, applied geophysics

## Abstract

This article analyses the architecture of the Early Iron Age royal burial mound Tunnug 1 in the “Siberian Valley of the Kings” in Tuva Republic, Russia. This large monument is paramount for the archaeological exploration of the early Scythian period in the Eurasian steppes, but environmental parameters make research on site difficult and require the application of a diversity of methods. We thus integrate WorldView-2 and ALOS-2 remote sensing data, geoelectric resistivity and geomagnetic survey results, photogrammetry-based DEMs, and ortho-photographs, as well as excavation in order to explore different aspects of the funerary architecture of this early nomadic monument. We find that the large royal tomb comprises of a complex internal structure of radial features and chambers, and a rich periphery of funerary and ritual structures. Geomagnetometry proved to be the most effective approach for a detailed evaluation of the funerary architecture in our case. The parallel application of several surveying methods is advisable since dataset comparison is indispensable for providing context.

## 1. Introduction

The Early Iron Age in the Eurasian steppes marks the beginning of the appearance of fully mobile pastoralist groups and a steeply hierarchical society with a social elite of warriors fighting from horseback [1,2,3,4]. The circumstances and reasons for its inception are the topic of a hot debate. For the first time in the history of the Eurasian continent, a largely coherent material culture spread from Mongolia to Eastern Europe within only a few generations [5,6]. The earliest remains of this archaeological culture—defined by the “Scythian triad”, consisting of an assemblage of horse gear, weapons, and items decorated in animal style [7,8]—are found in Tuva Republic, southern Siberia [9,10].

Large Early Iron Age burial mounds are plentiful on the river terraces of the Uyuk Valley in Tuva Republic, hence it has been dubbed the Siberian “Valley of the Kings” [11]. The area is widely assumed to have played key roles in the formation of Early Nomadic societies of the Eurasian steppes. Scythian material culture in Tuva is divided into three periods: An early Arzhan stage (9th/8th century BCE) followed by the Aldy-Bel stage (7th to 6th century BCE) leading into the Uyuk-Sagalyn period (5th to 3rd century BCE) [12]. Despite the archaeological documentation of many Iron Age burial mounds in Tuva, the large tomb Arzhan 1 remained the only archaeological monument dating to the formative period of “Scythian” material culture at the end of the 9th/beginning of the 8th century BCE and effectively defined an entire period by itself. In 2014, a much smaller burial mound with around 50 m diameter was excavated (Arzhan 5), which, through architectural and stylistic parallels of its objects, seemed to date to a similar period [13]. Only in 2017, a first survey at the royal burial mound Tunnug 1 established that this tomb was the earliest known so far. The monument was first analyzed through WorldView-2 data, before a field campaign generated a high-resolution 3D-model and recovered first samples of preserved wood [11].

**Figure 1:**
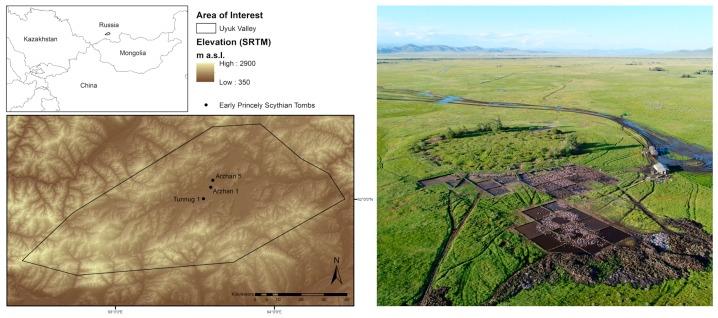
(**left**) Overview over the Uyuk Valley and the earliest known royal Scythian burial mounds in the Eurasian steppe mentioned in the text. (**right**) Aerial view of the burial mound Tunnug 1 with the 2018 excavation quadrants in the southern periphery (looking northeast) Photo: J. Blochin.

The key position Tunnug 1 takes within the chronology of the Early Iron Age steppes has since led to the establishment of a large excavation project. Due to the size of the burial mound (Figure 1) and its periphery it is difficult to get a first overview over the extensive funerary architectural complex and—even though necessary—excavation is slow and tedious under the Siberian environmental circumstances. The burial mound’s location within a swamp leads to irregular vegetation growth and an overall difficult readability of vegetation marks. Freezing soil, cryo-, and bioturbation complicate the stratigraphy. In order to generate an overview over the site and its surroundings it was thus necessary to integrate a number of data sources to generate a holistic picture of the buried funerary architecture. We were looking to answer the question whether we can detect additional peripheral monuments, later destructions of parts of the burial mound, or internal architectural structures.

## 2. Methods

A first excavation campaign in 2018 at the burial mound Tunnug 1 showed that the surface vegetation is a questionable indicator of subsurface architectural structures and needs to be approached with extreme care when trying to base excavation planning on it. Suboptimal decisions, in terms of which areas to excavate first, finally made it necessary to integrate several data sources in order to make decisions on a broader and more diverse basis taking into account the different qualities and characteristics of the methods described below. Usually, vegetation marks and topographical features are used as a first assessment of the underlying archaeology. From the first excavation area we understood that any archaeological features are lying relatively close to the surface. We started out with visually interpreting optical satellite data and conducting an amplitude analysis of synthetic aperture radar data of the site. Then we completed a drone survey of the burial mound and generated a high-resolution 3D digital elevation model through photogrammetry. We conducted a geophysical survey of the site employing geoelectric resistivity and geomagnetometry. Geomagnetometry in particular was chosen because it had been applied successfully in the periphery of other large burial mounds before by Fassbinder et al. [14,15]. Finally, archaeological excavation provided a validation approach for the non-invasive methods.

### 2.1. Visual Interpretation of WorldView-2 Data

The mound was first analyzed on WorldView-2 data which was provided through the DigitalGlobe Foundation. The data has a resolution of 0.46 m at nadir for the high-resolution panchromatic band. Also available are eight multispectral bands; consisting of four standard colors (red, green, blue, and near-infrared 1) and four new bands (coastal, yellow, red edge, and near-infrared 2) [16]. A visual interpretation has long been established as a tool for preliminary site analysis [17]. Interpreting the data seemed to show radial features which stand out against the background and led to the hypothesis that a part of the burial mound could have been constructed from wood in a wheel-like fashion with spokes extending from a central burial chamber. This reflected a similarity with the early Scythian royal tomb Arzhan 1 [9]. We created a series of false-color images which allowed us to differentiate between the burial mound and its surroundings as well as understanding which parts of the monument and its periphery are covered by denser vegetation and which parts represent open stone fields in which the water quickly drains and leaves little substrate for plant life. We used the following false color band combinations in order to highlight vegetation features and open stone areas; NIR_1_/Red/Green, NIR_1_/Green/Blue, NIR_1_/RedEdge/Red, and NIR_2_/Yellow/Coastal.

### 2.2. Multipolarization SAR Amplitude Data Analysis

ALOS-2 L-band SAR data acquired through the Japanese Space Agency (JAXA) [18] was used for a preliminary site analysis. Despite the rather low spatial resolution of ALOS-2, the longer L-band wavelength of the system allows for deeper penetration, offering unique advantages compared to the shorter wavelength systems. After radiometric calibration and multi-looking, the data was geometrically corrected to a world coordinate system based on the Range-Doppler approach [19] using the SRTM 1-arcsecond data as DEM. Due to the high dynamic range of SAR data, we performed a histogram analysis, before identifying the burial mound based on amplitude thresholding.

### 2.3. Drone Survey and Photogrammetry-Based Digital Elevation Model

A drone was flown over the burial mound in a regular flight pattern while shooting pictures with 70% overlap. A high-resolution 3D digital elevation model and ortho-photographs were then generated through a structure from motion approach with the software Agisoft [20]. The model allowed for accurate mapping of surface structures of the monument and slight topographical differences in the periphery.

### 2.4. Geoelectric Resistivity Survey

Geoelectric resistivity was chosen as a survey method because we knew from the preliminary excavations, that archaeological stone features were right under the surface and we thus expected good results. We used a Resistance Meter 85 with Multiplexer Unit, which controlled five mobile electrodes and two fixed electrodes. The five mobile electrodes, so called sample probes were arranged in a fixed distance of 0.5 m on a measuring device [21,22]. Measurements were taken at an interval of 0.5 m in the running direction in which the electrodes were controlled independently, resulting in a total of eight measurements at each location. This configuration made it possible to not only record the ground resistivity close to the surface, as with a dipole–dipole measurement, but also acquire measurements at deeper depths (up to 1.0–1.2 m) in coarser resolution. The general layout of the survey quadrants 30 × 30 m and oriented the same way as the excavation grid. This grid formed the basic unit for both geophysical prospection methods and could easily be extended by any number of grids into the cardinal directions. The data was then processed with the software Snuffler. The survey areas were geolocated using a total station in order to import the survey data into a geographical information system for further analyses.

### 2.5. Geomagnetic Survey

Iron Age burial mounds in the Eurasian steppe are known to have been locations for intensive ritual activity including rituals connected to feasting and fire. The magnetometer survey was carried out using a Geometric 858 magnetometer in the so called duo-sensor configuration [22,23]. The two cesium sensors were fixed parallel on a special light-weight carrying device similar to [14,15]. The distance between the two sensors on the device was 0.5 m, amounting to a line width of 1 m. The survey areas were measured in zigzag mode, with an orientation measurement every five meters in order to locate the constant measurements relative to the line. The data was process in the software Magmap 2000 and the software Surfer for data enhancement and visualization. As with geoelectrics, the survey areas of the geomagnetic measurement were then located with a total station in order to integrate the visualized results into a geographical information system.

### 2.6. Excavation

The excavation method was adapted to the complex mixed layers which are a result of almost three millennia of cryo- and bioturbation as well as alluvial erosion. We first removed the top soil and cleaned the identifiable stone structures. Then an organic silt–clay layer of around 20 cm was removed. This second step already concluded the excavation in most areas in the periphery, since a clean alluvial layer without cultural artifacts was identified underneath. As a third step stone structures were taken apart while documenting their stratigraphic profiles. The documentation of the excavation process was carried out using photogrammetry. After each stage of excavation, every quadrant was documented with UAVs (DJI Phantom 4 Advanced/3 Advanced), from these sets of photographs 3d-models, elevation models and ortho-photomaps of every quadrant were built. Object drawings were created in Autodesk AutoCAD using data from the photogrammetry. All artifacts found during excavations (animal bones, ceramics fragments, iron, bronze, human bones, and stone products), as well as wood, soil, or bone samples taken for analysis were recorded using a total station.

## 3. Results

We processed the different data sets in the geoinformation software ArcMap 10.4 (Esri, Redlands, CA, USA) as well as in AutoCAD 2018 (Autodesk, San Rafael, CA, USA). Only selected sets are shown for illustration and orientation purposes. The results of the different data sets are elaborated upon below. The coordinates in the images are left out on purpose since the authors do not want to promote site destruction and looting which are known to be a problem for this type of sites [24].

### 3.1. Visual Interpretation of WorldView-2 Data

**Figure 2:**
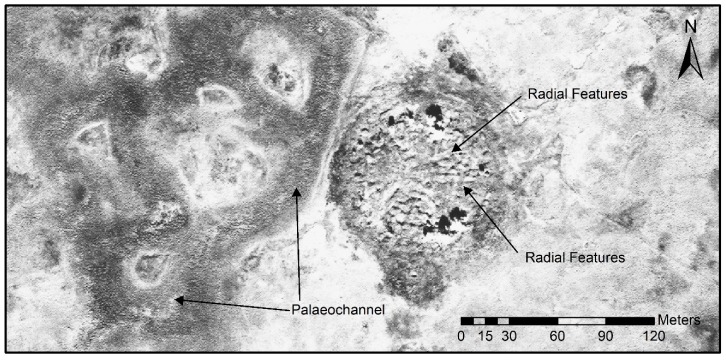
High-resolution WorldView-2 data (panchromatic band with 0.46-m resolution at nadir) of the site clearly shows later paleochannels which impacted the western border of the burial mound. On the burial mound, vague radial features can be identified.

The high-resolution WorldView-2 data was an excellent starting point for a preliminary analysis of the burial mound and its surroundings (Figure 2). The rugged surface structure of the stone mound can already be seen in the panchromatic band with 0.46 m resolution. Tall-standing birch trees which grow on the site cast shadows and obstruct the view of important parts of the mound in the north and the south. Few peripheral archaeological structures can be seen. A larger stone mound in the south with a central pit is the most obvious one. The rest of the periphery shows little structure except for some naturally caused flow-like marks. In the west of the monument, very clear paleochannels can be seen. The signature is caused by vegetation growing lusher on the ancient river bed, since it still carries a lot more water than the area south and east of the burial mound.

### 3.2. Multipolarization SAR Amplitude Analysis

**Figure 3:**
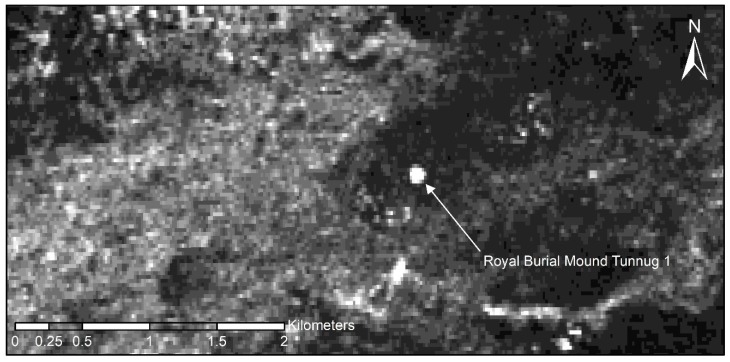
ALOS-2 data (HV polarization) showing clearly that in the larger vicinity of the mound no monuments of equal size and composition can be found.

Despite the rather coarse resolution of approximately 4 × 4 m, the burial mount Tunnug 1 was clearly identifiable in both HV (horizontal transmit and vertical receive) and HH (horizontal transmit and horizontal receive) polarization. The composition of the stones in the burial mound lead to a relative strong backscattering in HH, probably also due to occurring double-bounce scattering, as well as a strong backscattering in HV, as the differently oriented stones will cause a shift in the polarization. Interestingly, Tunnug 1 is much better discernable in the ALOS-2 L-band data, than in other short wavelength data (Figure 3). This is due to the dominance of the backscattering from the short vegetation on top of the mound in X-band, which only slightly differs from the surroundings, while the L-band penetrates this short vegetation and a limited part of the soil surface, so that the stone structure becomes visible. Analyzing the ALOS-2 data, it appears that in the close vicinity of the monument there are no other funerary architectural structures of similar size and composition.

### 3.3. Drone Survey and Photogrammetry-Based Digital Elevation Model

**Figure 4:**
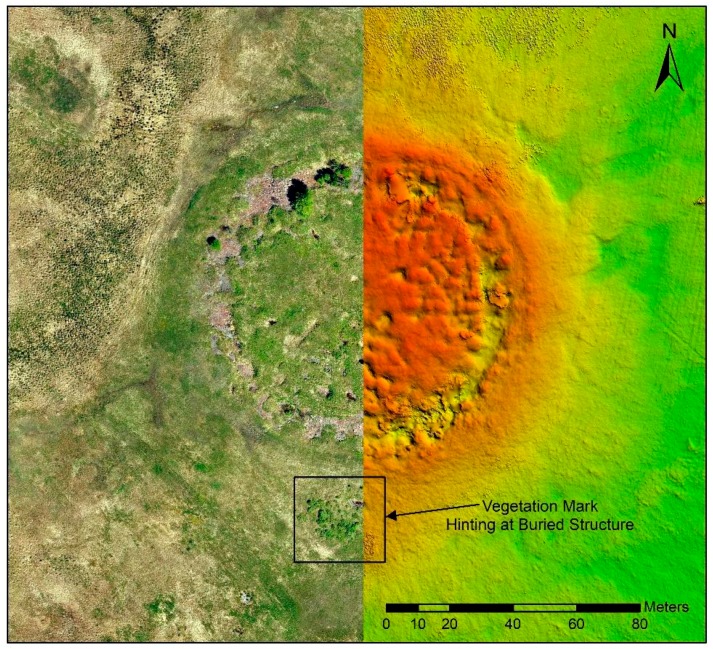
(**left**) Ortho-photo generated from 80 individual pictures captured by an unmanned aerial vehicle (UAV), approximate resolution 5 cm. (**right**) Digital elevation model generated with an approximate resolution of 15 cm. Note the clear vegetation mark in the southern periphery of the burial mound indicating a buried structure.

During the first preliminary field campaign in 2017, we generated a high-resolution digital elevation model (Figure 4) by means of a structure from motion approach with the software Agisoft. A drone was flown over the burial mound at a height of 50 m taking 12 MP pictures with a 70% overlap and a ground sample distance of 3.5 cm/pixel. Based on these data we then combined 80 individual images into an ortho-photograph. Both datasets give a very clear impression of the topographic and vegetation characteristics of the site. The near surroundings of the burial mound are quite uniformly grown over. Only in the southern periphery, an accumulation of small bushes in approximate circular shape hinted towards a buried structure.

### 3.4. Geoelectric Resistivity Survey

**Figure 5:**
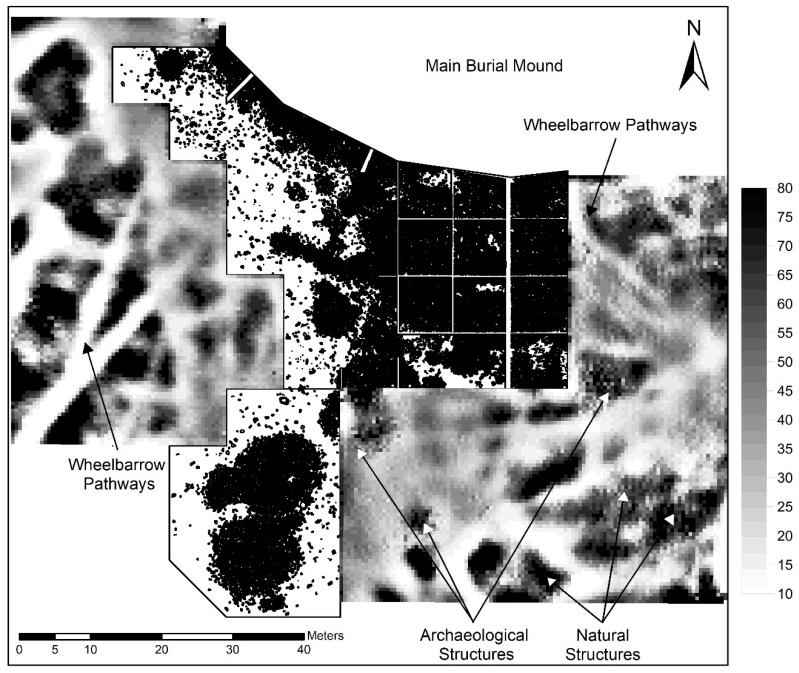
Results of the geoelectric resistivity survey (in Ω displayed in grayscale) and the plan of the 2018 excavation (in black and white) in the southern periphery of the main burial mound. Archaeological and natural structures show a very similar signature and can be confused easily. Wheelbarrow traces show the most obvious marks.

The geoelectric resistivity survey showed mixed results, and thus was only used selectively with a total survey area of 6500 m^2^. The raw data of the geoelectric survey covers a value spectrum of 14 to 80 Ω. We were able to identify some structures because they happened to show up in the profile of the 2018 excavation. Others remained unrecognized. Many natural features showed very similar signatures as the archaeological structures and therefore it was difficult to read the data. Interestingly, the geoelectric resistivity survey very clearly depicted the narrow paths which were used by workers on the excavation to transport stones and soil to the backdirt deposit (Figure 5). The frequent usage of these paths over the course of three months in summer 2018 likely led to a densification of the ground changing its properties and affecting its conductibility [21]. Due to the many false positives, the geoelectric resistivity survey was only carried out in a limited part of the southern, western, and northern periphery. On the main burial mound which is largely covered with a thick layer of stones, it was impossible to get the probes into the ground and therefore the survey stopped at the edge of the monument. Due to the relatively thin topsoil above the archaeological stone structures, we expected no better results, even if the high moisture content of the soil could have made the acquisition of archaeologically relevant data possible.

### 3.5. Geomagnetic Survey

**Figure 6:**
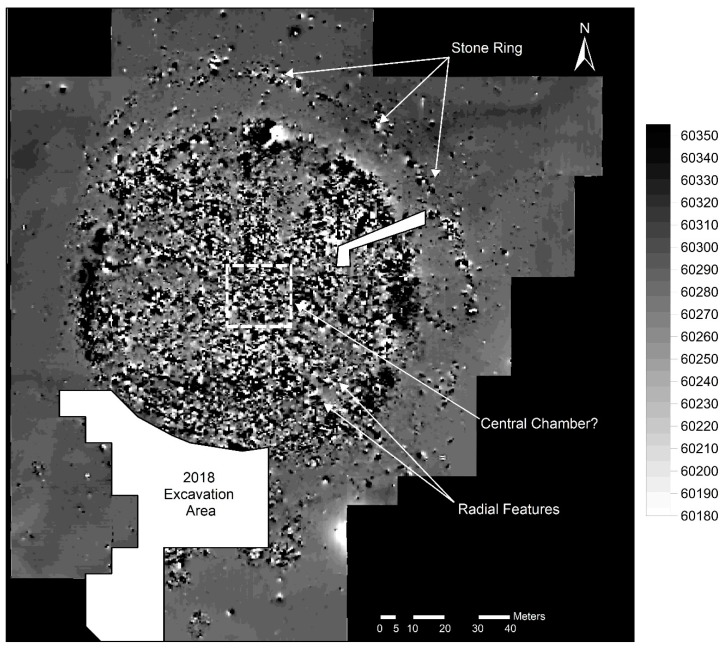
Results of the geomagnetic survey (in nT) with the clearly visible outer stone ring of the burial mound, additional unknown peripheral structures in the south, clear internal radial division of the tomb and a potential central chamber.

In total, an area of approximately 27,000 m^2^ was covered by the geomagnetic survey. The geomagnetic survey showed very good results under the conditions on site (Figure 6). Anthropogenically placed stones displayed a high contrast against the natural background. This is a very interesting effect since fired or otherwise processed materials (e.g., bricks) are generally absent on site. The composition of the stones will be analyzed in a future field campaign. Based on their reddish patina, they might have a high Fe content. The main result is a very clear stone ring around the monument which is not visible in the WorldView-2 data and neither in the high-resolution ortho-photography, due to the high ground vegetation. The earth’s magnetic field had an average value of 60,290 nT at the time of the campaign. The data, processed with a low-pass filter and reduced by a coarse-grained moving average filter, show only minimal superposition by (interfering) bipolar anomalies compared to the raw data. This processing made it possible to sharpen the geomagnetic measurements in the area of potential chambers. Walking on site, some of the stone accumulations of the circle can be identified, but the extent remained unclear even when mapping out all visible stones with a total station. The outer ring of stones extends from the northwest to the southeast of the burial mound. In the northwest it is broken by the paleochannel, likely eroded and carried away by the river. In the south we can see a number of other peripheral archaeological structures which might have made use of the stones from the central burial mound and the surrounding circle thus destroying parts of the site through sourcing building material. Despite difficulties due to surface roughness, we were able to cover the entire burial mound with the geomagnetic survey. It turns out that the radial features already seen in the satellite imagery are even more clearly visible in the geomagnetometry results. The burial mound seems to be separated into sectors running from the outside to the inside. Not all sectors have the same size. In the northwest the radial features are denser whereas in the north and west, the sectors show with great clarity. A very deep trench at the border of the burial mound is rendered in black. Its existence was already apparent from the excavation in 2018 but it might be the case that this circular ditch does not run through along the entire border of the burial mound. In the south internal structures of the burial mound are unclear and the image is very noisy. Simple filtering was applied, but without decent results. However, the center of the burial mound seems to contain another structure. Rectangular in shape and roughly 18 m × 19 m it might be that the magnetometry survey was able to detect a large central burial chamber.

Additional peripheral archaeological structures were discovered south of the main burial mound. We were also able to define the extent of a large amorphous structure which was not completely excavated in 2018. We also surveyed the areas of the 2018 excavation where some stones had been left in place. The stones showed again with great clarity. The limited depth of the topsoil allowed for a similarly distinct imaging of the stones as if they would be lying out in the open.

### 3.6. Excavation

A carefully documented excavation obviously will always yield the most accurate results and it is not in the scope of this paper to elaborate on the details of excavation. Interested readers may refer to a preliminary report published by Sadykov et al. in 2019 [25]. Excavation is expensive and comparatively slow, especially on this site where extreme temperatures (day–night amplitudes of over 40 degrees are a regular occurrence), flooding, and until late May, snowstorms can occur.

**Figure 7:**
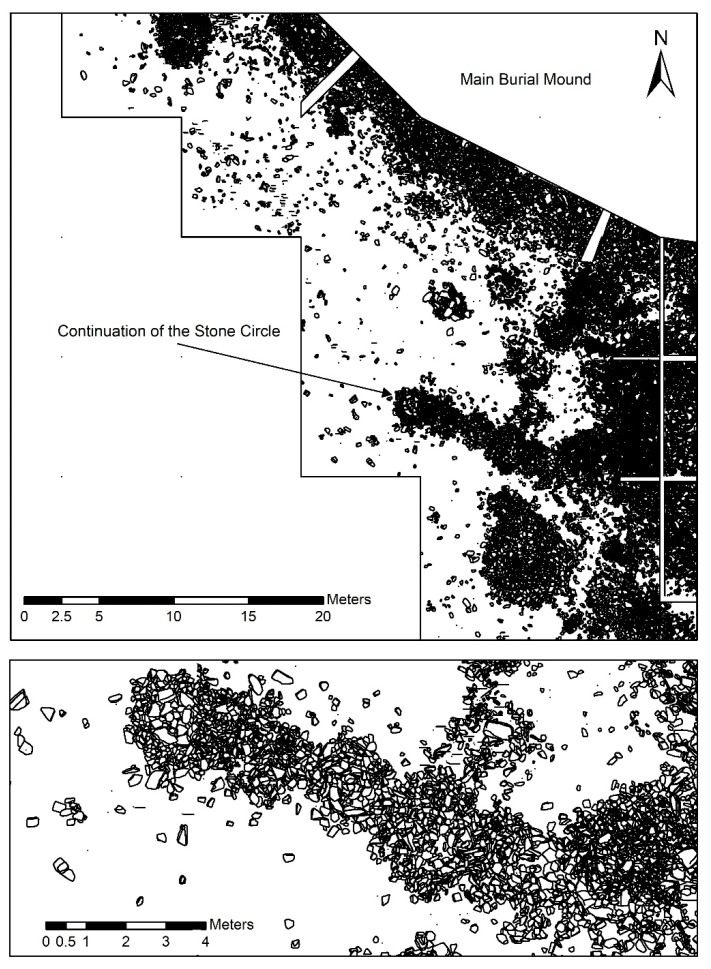
(**top**) Excerpt of the 2018 excavation report showing the documented stone structures underneath an only 20 cm deep topsoil layer in the southern periphery of the burial mound. (**bottom**) Close-up of a line of circular shaped stone mounds of ~2 m in diameter lined up in approximate east–west direction. Through combination with the results of the geomagnetic survey with the excavation documentation, we can date these stone structures to the time of the construction of the main burial mound.

The 2018 excavation campaign carefully documented every stone with a diameter >10 cm and produced accurate stone-by-stone drawings from ortho-photos in AutoCAD (Figure 7). Not all peripheral archaeological structures are easily datable, since sometimes there are neither carbon-rich materials nor archaeological objects associated with them. Such a case presents a string of small stone platforms adjacent to each other in the southwestern periphery of the burial mound. The geomagnetometry survey and the discovery of the outer stone ring around the central burial mound allowed assigning these small stone structures to the ring due to their matching distance from the border of the main burial mound.

## 4. Discussion

In 2017, before the first preliminary survey in the “Siberian Valley of the Kings”, WorldView-2 data and ALOS-2 synthetic aperture radar data were employed to gain first insights into the site and its surroundings. Despite its coarse resolution the radar dataset proved extremely effective in determining if other large burial mounds could be found in the vicinity of the Tunnug 1 site. The vegetation in the flood plain is difficult to read and high-resolution optical satellite data shows many features of approximate circular shape which is of natural origin—e.g., created through sinuosities. WorldView-2 data on the other hand was instrumental for understanding the immediate surroundings of the site. Through creating false-color images we were able to neatly define the outline of the paleochannels to the west of the site [25,26]. With a resolution of under half a meter of the panchromatic WorldView-2 data, it is even possible to understand basic internal architectural features of the royal tomb such as the radial features which delivered well-preserved wood for radiocarbon dating [11].

During the 2018 excavation campaign no means for extensive geophysical studies were available. We thus resorted to interpreting differences in vegetation and topographic features for the planning of the excavation. For this purpose we had generated a high-resolution digital elevation model and ortho-photographs. The approach had worked well in the case of the first preliminary study in 2017 [11] where we quickly located datable material. However, in 2018 when we had to choose an area in the periphery, we ended up selecting a circular feature comprising of several low bushes and interpreted it as a smaller burial mound south of the main monument (Figure 4). This turned out to be a mistake which would cost us a lot of time since we began to uncover a part of a large amorphous stone structure which could not be completed in one excavation campaign. In the further course of the excavation we learnt that at the royal burial site Tunnug 1 seemingly obvious vegetation marks are often misleading.

**Figure 8:**
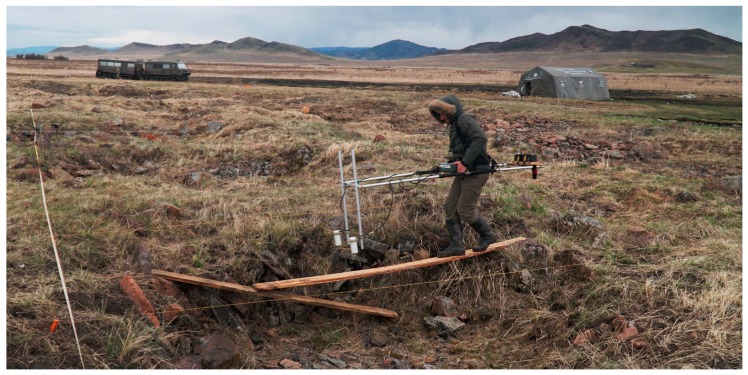
Wooden planks and a light weight geomagnetometry device made the survey on the burial mound possible. Ground-penetrating radar and geoelectric resistivity would not have been possible under these circumstances. Photo: T. Wallace.

In the 2019 precampaign survey we opted for employing geomagnetometry and resistivity in order to better define the excavation areas and the extent of the site. The results of the geomagnetometry allowed us to confirm the existence of a stone ring around the royal burial mound. The magnetometer accurately indicated anthropogenic subsurface stone structures. At the edge of the 2018 excavation zone we were able to understand where stone structures lead into the profile and thus could derive expectations as to where we should see results. The magnetometer showed the expected results consistently. Predefined areas in which we would expect no archaeological structures (e.g., near the paleochannel to the west of the burial mound) also remained consistently empty. It appears that the stone ring around the burial mound used to be complete at the time of the construction of the tomb. During later times it was first impacted in the south through the construction of a large amorphous structure dating to the 2nd–4th century AD [27] and later completely destroyed in the west through a change in the course of the river, leading to the creation of the paleochannel visible in the WorldView-2 data (Figure 2). It was even possible to assign a row of small circularly shaped peripheral stone structures to the ring. These structures were excavated in 2018 but could not be dated or assigned to a particular material culture since they neither yielded carbon-rich material nor archaeological artifacts. The distance of these structures from the edge of the main burial mound of around 15 m is consistent with the distance of the stone ring around the burial mound. We can expect the stone ring to consist of many such small circular stone structures. Given the light weight of the custom made geomagnetometry equipment, we were able to bridge deep pits (possibly caused by collapsing chambers) on the burial mound itself and survey the rugged terrain (Figure 8). Both ground-penetrating radar and geoelectric resistivity would not have been a valid choice under these circumstances (Figure 8). This part of the survey allowed for insights into the internal structure of the royal burial mound and made it possible to identify the vague radial features seen in WorldView-2 data and the digital elevation model more closely. The burial mound clearly is subdivided into uneven sectors possibly through a construction of larch logs similar to Arzhan 1 [9] and Arzhan 5 [13]. In the middle of the mound a large rectangular feature was detected, which might be a central burial chamber. The dimensions of 18 m × 19 m are exceptionally large considering that the outer wood construction of the central burial chamber at Arzhan 1 measured roughly 10 m × 10 m [9].

The geoelectric resistivity, however, did not work very well in our case. A reason for this might be that the ground is usually very moist and thus very conductive [21], which could lead to the current flowing relatively freely even in areas with a lot of stones. What seemed to make a difference is the density of the soil. In areas where the soil had been compressed by the pathways wheelbarrows were taking to the backdirt deposition area, we received very clear signatures, which of course were not particularly helpful in gathering archaeological data. In one case we also detected the burrow of a badger and thus were able to identify an additional source for bioturbation on site. The heterogeneity of the data and the similarity of natural and anthropogenic features made geoelectric resistivity on our site a method which—applied without the context of other data—might lead to numerous false positive detections of archaeological structures. An additional shortcoming was that due to the need of planting electrodes in the ground for close-fitting contact, the method could not be applied on the burial mound itself.

## 5. Conclusions

Only the application of a diversity of surveying methods produces a reliable and holistic image of complex royal burial site like Tunnug 1 in the “Siberian Valley of the Kings”. The complex vegetation patterns, cryo- and bioturbation, and modern human impact can easily lead to misinterpretations and suboptimal decision making on site. Surveying the wider periphery of the Tunnug 1 site with ALOS-2 and WorldView-2 data contextualizes the monument with its environmental surroundings. The promising SAR approach will be expanded in future campaigns [28,29]. UAV- and photogrammetry-based surveying methods in combination with geomagnetometry and geoelectric resistivity measurements allow for deeper insights into the complex ritual and funerary architecture of the royal burial mound Tunnug 1 and its periphery. The magnetometry survey led to the discovery of additional stone structures in the southern periphery, a well-constructed circular ring consisting of dozens of smaller round stone platforms around the main burial mound, and most importantly it provided first insights into the internal architecture of the tomb. A central rectangular burial chamber seems to connect to radial features which split the monument into uneven sectors. Under the rules of the Russian Cultural Heritage Administration it is mandatory to completely excavate a site. However, for areas where these rules do not apply, remote sensing as well as geophysics are employed as a mean to gather information and interpret the data without further excavation; it is paramount that at least two methods are used in conjunction. It is very difficult to predict which method will yield the best results and thus the parallel application of several methods increases the chance for success and provides a basis for mutual evaluation if the practitioner has access and funds for such an approach. A reasonable extension of the surveying activity on the burial mound would be electrical resistivity tomography in order to generate an approximate profile [30] of the burial mound and understand where chambers might lie. However, the mound is built up from a thick stone package and it can be very difficult get good results under these conditions. Additional information comes at a cost and thus has to be evaluated against its usefulness. Both magnetometry and resistivity have not been applied much on stone burial mounds due to surface roughness, but they are known to work with burial mounds made up of soil [31,32]. It remains to conclude that in every case in which we derived a meaningful archaeological interpretation, it was at least rooted in the combination of two different datasets. The integration of different datasets not only makes interpretations more secure, in many cases it enables them in the first place.
